# The significance of gratitude for palliative care professionals: a mixed method protocol

**DOI:** 10.1186/s12904-019-0412-y

**Published:** 2019-03-21

**Authors:** Maria Aparicio, Carlos Centeno, María Arantzamendi

**Affiliations:** 1 Universidad de Navarra, ICS, ATLANTES, Campus Universitario, 31080, Pamplona, Spain; 2 Palliative Care Clinical Nurse Specialist at St Christopher’s Hospice, London, UK; 3 Clínica Universidad de Navarra, Servicio de cuidados paliativos, Av. Pio XII, 31008 Pamplona, Spain; 4 IdiSNA, Instituto de Investigación Sanitaria de Navarra, Pamplona, Spain

**Keywords:** Gratitude, Meaningful recognition, Palliative care, Health care professionals, Mixed methods, Motivation, Patient, Relatives, Burnout, Compassion fatigue

## Abstract

**Background:**

In palliative care (PC) patients and relatives (P/R) often show their gratitude to the healthcare professionals (HP) who care for them. HP appreciate these displays of gratitude, although the impact of the same has not been examined in detail. Publications analysed tell personal experiences in which HP say that displays of gratitude create sensations of well-being, pride and increased motivation to carry on caring. No systematic examination in PC was found. These aspects related to gratitude may be important in the field of PC, where there is constant exposure to suffering and the preoccupation which arises from wanting to help HP to go on with their work, but it needs closer study and systemisation. The purpose of this study is to understand the significance and the role of the gratitude received from P/R for palliative care health professionals (PCHP).

**Methods:**

A suitable mixed method will be used. The first phase will be quantitative and will consist of a survey, piloted by experts, whose goal is to explore the current situation in Spain as regards displays of gratitude received by HP at PC services. It will be sent by e-mail. The results from this part will be incorporated into the second part which will be qualitative and whose goal is to understand the significance of the experience of receiving displays of gratitude from the perspective of PCHP, using a phenomenological approach. Interviews will be undertaken amongst PCHP. The interview guide will be designed after taking the survey results into account. The project has been granted ethical approval.

**Discussion:**

These results are set to provide a key contribution within the context of the growing preoccupation on how to care for HP, how to ensure retention and keep them from resigning, as well as preventing burnout, emotional fatigue and boosting their resilience. In order to do this, it is both interesting and ground breaking, to analyse the repercussion of spontaneous gratitude shown by P/R towards PCHP, to see if this is a useful resource to reduce these problems and to encourage the greater presence of dignity and humanisation, for both those receiving care and for those providing it. This gratitude may be one of these strategies.

**Electronic supplementary material:**

The online version of this article (10.1186/s12904-019-0412-y) contains supplementary material, which is available to authorized users.

## Background

In Palliative Care (PC) it is commonplace for patients and relatives (P/R) to be grateful for the care received. Gratitude arises as a reaction to something which is appreciated or whose results are positive [1]. It occurs due to the value given to an experience [2], or as an emotional response to something which has happened to us [3]. Balduin Schwarz considers it to be an emotion in which the human being, in a meaningful way, spontaneously reacts to something initially given to him or her [3].

In the recent literature gratitude and its benefits have had more attention but the phenomenon of gratitude remains conceptually confusing [4]. It is characterised as a stable personality trait, a transient emotional state, and a moral virtue, or as a coping response and a life orientation [1, 5–7].

It has been intuitively linked to happiness and wellbeing and inversely linked with negative emotions such as anger, anxiety or envy [8]. Moreover, grateful people tend to be satisfied with what they have and are so less susceptible to emotions such as disappointment, regret and frustration [8]. Most of the research regarding gratitude conceives it as a trait, so focuses on the effect of being grateful or practising gratitude.

There are studies describing the benefits of conducting regular actions of expressing gratitude on psychological and physical well-being [6, 7, 9–12]. Others suggest that gratitude helps to lessen stress [13–15] and even anxiety when facing death [16]. As well, feeling gratitude is associated with increased job satisfaction and productivity, fewer absences and decreased burn out [17–20].

In addition, gratitude encourages other positive emotions and the ability to cope during hard times [21]. Which may be valuable, above all, in more demanding work contexts such as PC, intensive care and the emergency department, where HP work in vulnerable situations, accompanying P/R during suffering and at life’s end and where they tend to suffer negative effects since they are exposed to complex situations of care and suffering.

Practising gratitude seems to be beneficial, but little is known about the impact of receiving gratitude, understood as a transient emotional state. A recently published scoping review about gratitude displays from P/R towards HP shows that there is mainly anecdotal evidence regarding it [4]. The review suggests that receiving expressions of gratitude may have an important personal and professional effect on HP who experience emotions, such as satisfaction [22–25], wellbeing [22, 26] and gratitude [22–25]. Demonstrations of gratitude are also perceived as an incentive to carry on working with the same dedication, love, efficacy and effort [25, 27] and even as a source of motivation and encouragement [23, 25, 27] to continue caring for others [25, 27], leading to greater life satisfaction [7]. One of the two research based studies included in the review points out that within a therapeutic relationship when the emergency and oncology nurses perceive gratitude from their patients there is a decrease in their burnout [28], specifically with emotional exhaustion and personal accomplishment, but not with depersonalization [28].

In the context of PC, burnout is worrying. Rates of burnout identified in PC vary from between 25% [29, 30] and 62% [31], although some studies suggest that there are no significant differences in degrees of burnout in PC HP compared with other HP [32, 33].

Related to burnout in PC there have been recent mentions and further study of other concepts such as compassion fatigue, which differs from the above to the extent that compassion fatigue is a form of burnout specific to helping HP [34]. They conclude that both concepts or phenomena present a positive correlation [19, 20, 35, 36]. In spite of a recent suggestion which critically re-examined the term compassion fatigue in favour of a new discourse on healthcare provider work-related stress [37].

HP have different coping strategies for the stress caused by working in this area [19, 20, 35, 36], and are thus able to ensure the standard of practice with compassion, satisfaction and resilience. Individual experiences seen in the literature suggest that displays of gratitude can be a source of meaningful recognition. Meaningful recognition involves acknowledging one’s behaviour and the impact their actions had on others [38]. This is what P/R do when expressing gratitude to their HP. Meaningful recognition is one of the six elements associated with healthy work environments [39], which seems to suggest that displays of gratitude may have a positive and protective effect on working in situations of suffering. The literature review is in line with this [4]. It points out that expressions of gratitude from P/R are valuable for HP and have an effect on them and highlights that receiving gratitude is an opportunity for HP to reflect, which can be capitalized on for positive development [4]. Also, support the idea that gratitude has a positive effect on the person who receives it [1]. This hypothesis needs further consideration with a systematic approach.

P/R express this gratitude for care received in different ways. For example: words of. thanks orally, or written in letters, a variety of gifts such as chocolates, flowers, etc. which are given to the HP. While it is common for P/R to express gratitude in the context of care [40–48], even in other settings and not only in PC [27, 28, 41–43], there is limited research base evidence on the effect that gratitude displays have on palliative care health professionals (PCHP).

There is evidence that HP place special value on P/F displays of gratitude [25, 49–52]. Some HP even file them away and describe them as a personal source of motivation [50]. For all the above reasons, this study has been proposed to cover the gap in the literature regarding a topic as important as this, with the aim of understanding the significance and the role which gratitude received from P/R has for PCHP.

The specific objectives of the study are to:

- Describe displays of gratitude received by PCHP
- Understand the significance for PCHP of displays of gratitude
- Understand the possible repercussion of gratitude on HP themselves, their well-being, motivation and job satisfaction.

## Methods and design

Mixed method will be used. This methodology is useful when studying complex phenomena about which little is known and both quantitative and qualitative methods are needed to tackle the complexity of the phenomenon, or when the results of one method favourably condition the second [53], aspects which arise in this study. The combination of the quantitative and the qualitative parts help better understand the phenomenon [54].

An explanatory sequential design will be used, as Creswell suggests as adequate for relatively new fields on which the quantitative results are further explained and interpreted with the qualitative data [53, 55, 56]. The first phase will be quantitative - a survey – to collect data from a larger group. This allows obtaining an overview of the topic (gratitude displays from P/F in PC) and identifying possible rich informants from all the PC services, for the next data collection. The second phase will be qualitative – interviews - allowing to obtain a deeper insight into the topic. The results of the first part will be taken into consideration and integrated into the development of the latter. The results will also be integrated into the discussion on results.

The qualitative part will take priority because we are interested mainly in gaining an in-depth understanding of the value which experiencing gratitude has for PCHP. Regarding the theoretical perspective, a pragmatist point of view will be applied, using different methodological approaches for the particular research problem under study [57]. Full integration will take place when data collection and the analysis of the different components of the study are complete. Then the quantitative findings will be considered and linked with the qualitative data. This is similar to what Moran-Ellis et al. (2006) termed “following a thread”, the idea of following the thread of an issue through different data sets [58].

### First phase: Quantitative, survey for PCHP

#### Goal

The goal of this part of the study is to explore the current situation in Spain as regards demonstrations of gratitude received by HP who work specifically at PC services.

#### Population and sample size calculation

The target group is PCHP specifically dedicated to PC. There are some teams that attend PC patients and another kind of no PC patients, but these were not the focus of the study as their challenges and situation are different [59].

In Spain, PC development has been gradual covering all levels of attention as hospitals, residential and community [60]. In general, it is estimated that there is 1 resource of PC for 102.026 inhabitants, although it varies depending on regions. The PC coverage is through different types of resources: PC units, PC support teams in the community, PC support team in the hospital, PC mixed supports teams (community and hospital). There are also paediatric PC units and support teams. PC services include different HP, sometimes have the basic structure of doctors and nurses and others a more complete one including social worker, psychologist, physiotherapists and others [59].

In the absence of a census of PCHP the PC service directory is considered the best option for the study. The directory is developed by the Spanish Society for Palliative Care [60]. Its latest version is from 2015 and lists all the services of PC known in Spain and has an email of contact for each of these services [60]. This contact usually is the head, the lead or the manager of the team as well as the one who kind of represents the whole team. Expressions of gratitude often are addressed to them even if there are for the whole team, so is a rich informant about gratitude. It is decided to include all the services in the list to enhance representativity [61]. The sample will be made up of one member from each service.

In case a particular service does not have an e-mail address, contact will be made by telephone to obtain the email and thus complete the list to the greatest possible degree [62].

The sample will include representatives of all the PC services in Spain (*n* = 284), and as such, the total sample will be used. This is a scientific survey method to allow the drawing of robust conclusions for the whole population [63], especially on an area where there is no available data such as proportion expected or relevant minimum effect; previously published to calculate sample size [61, 62, 64].

The experience of gratitude is an area of limited knowledge, with no published information about its frequency, impact, and effect. So as it is difficult to estimate the sample size needed, a total sample approach was chosen. Having a total sample size of 284 will allow a robust exploration of the phenomenon of study.

#### Data collection

A survey will be carried out, using a systematic nationwide data gathering method [62]. A self-administered internet-based web survey will be sent out to collect data. The advantages of using this type of instrument include the speed of sending and collecting data, lower costs, better distribution and increased time for the respondent to answer, thus favouring longer answers to open questions [65–67]. On the other hand, there are a number of drawbacks: lower response rates, coverage error, lower degree of attention when reading, as it is computer-based, difficulties with internet access, difficulty in finding a representative sample [65–67]. For these disadvantages mentioned, such as the lower response rate we plan a follow up that was done following a literature review of how to increase rates and an incentive. The concerns regarding the access to Internet are not applicable since internet, the use of the email and computers are common working tools for PCHP. As mentioned earlier all the PC services of the directory are included to promote representativity. Therefore, the internet questionnaire will be used in this first part of the study.

#### Questionnaire design

The questionnaire was designed after an exhaustive review of the literature, which examines the experiences of gratitude received during professional practice, including publications of personal narrations, taking into account the lack of a systematic research approach.

The questionnaire is divided into three main parts. The former examines the experience of gratitude when caring in a PC service, with questions which probe specific displays of gratitude and their characteristics, how often they occur, who is being thanked, when, the use of the same within the workplace and the feelings they provoke. The second examines personal experiences of gratitude from P/R to HP with questions about the significance they hold for him / her and the degree to which they agree with different statements on the role of gratitude in: professional satisfaction, on improving one’s state of mind, on acting as a source of support in difficult moments, on their role as a reward for work done, on protecting against compassion fatigue, in preventing burnout, etc. The latter explores the socio-demographic characteristics of the sample. In total, the questionnaire consists of 7 closed questions to answer on the Likert scale, 2 open questions and 10 questions to characterise the sample (Additional file 1). The questionnaire design development is conducted considering evidence-based suggestions to create an attractive presentation, which helps to improve response rate and detailed filling in of data [62, 68].

Once the questionnaire is completed, the respondents are automatically asked if they wish to continue collaborating in the second part of the study, by taking part in an interview. If they accept, they will be asked for their name, e-mail, telephone number and the best time to call. This is decided to open the possibility of identifying possible participants for the second part of the study, beyond PCHP that we know they are interested on the topic.

#### Pilot study

The provisional questionnaire was evaluated by experts on PC and surveys [69]. The experts received the survey and an evaluation sheet, where they provided feedback on those points they considered relevant. They were also asked about clarity, comprehension and writing quality, related to: the subject being studied, the aim, questions, response choices, instructions given to the respondent when completing the task, as well as which questions might be withdrawn or added and the time needed to complete the questionnaire. Once the questionnaire had been adjusted, incorporating the suggestions of the experts, it was then sent on to a smaller group of clinical PCHP for final verification, who reported minor grammar mistakes that were corrected.

#### Survey process

The questionnaire will be sent to the e-mail contact at each PC service, using the survey platform Survey Monkey®.

The response rate to surveys varies in relation to the type of questionnaire, the aims and format as regards administration [70]. In self-administered web-based questionnaires, the estimated response rate is between 24% [71] and 42% [72].

Since the representativeness of the results, depends not only on the sample but also on response rates, available evidence has been used to design a plan to stimulate a higher rate of response, including:

- A personal e-mail or cover letter indicating that a questionnaire will be sent shortly [65, 68, 73–75] including the topic, goals and the study information sheet.
- The identification of the study’s authors will be stated in the e-mail together with the promise to inform them of the principal results [76].
- Identification of the institution sponsoring the study, including its logo [74, 76].
- A personal invitation to take part in the study, including the link to the questionnaire [68].
- The questionnaire is sent at the beginning of the week, avoiding vacation periods [75].
- Follow up with weekly reminders [62, 68, 73] for 3 weeks, only for those people who have not completed the questionnaire [73, 75]. This will be done automatically set up to do it at the survey monkey platform, without the need to screen who has answered or not.
- A thank you e-mail for those who have completed the questionnaire [62, 68, 73–75].
- Offers of incentives [62, 65, 68, 74]. In this instance, the incentive offered to one of the participants is enrolment on a research course provided by the Instituto Cultura y Sociedad of the Universidad de Navarra. As well as literature indicating that incentives help increase response rates, it also rewards the time dedicated to completing the questionnaire. This incentive will be mentioned in the informative e-mail and the raffle will be held once everyone has completed the survey. The winner will be contacted by one of the members of the research team.

As such, the following guidelines for monitoring and administration have been drawn up (Table 1).

**Table 1:** Protocol for survey follow up

Week 1	An advisory e-mail is sent to the whole sample with a personal invitation, advising them that they will soon receive a questionnaire, together with an information sheet on the study.
Week 2	A personal e-mail with a link to the questionnaire is sent to the whole sample.
Week 3	Follow Up 1: The first reminder with a link to the questionnaire is sent to all those who have not replied or have only partially replied.
Week 4	Follow Up 2: A second reminder with a link to the questionnaire is sent to all those who have not replied or have only partially replied.
Week 5	Follow Up 3: The third reminder with a link to the questionnaire is sent to all those who have not replied or have only partially replied.
Week 6	A personal e-mail thanking them for their participation is sent to all those who have completed the questionnaire fully.

Should the response rate be less than 55–60%, the contact details of the non-respondent services will be checked with a phone call to the service to update the contact details. Then the questionnaire will be sent again using updated data for these services and following the protocol mentioned above except for the invitation mail and the third reminder.

#### Data analysis

Data obtained will be analysed using descriptive analysis, with a measure of central tendency and frequency distribution measurements, as well as the possible relationships between the variables being studied.

The analysis and interpretation of this part of the study will provide information on data collection and analysis gathered in the second part of the study, [54] the qualitative.

### Second phase: Qualitative, Interview with PCHP

As previously mentioned, the survey’s results will offer data on displays of gratitude in PC and its characteristics, as well as on HP experiences in this respect and on the degree to which the expressions of gratitude are relevant for HP. This will provide an initial insight into the phenomenon and on HP experiences, which might be dealt with in depth during the interviews. Furthermore, it will allow the identification of possible participants in that part of the study.

#### Goal

The aim of the qualitative part of the study is to understand the significance of receiving displays of gratitude from the perspective of PCHP and to do this, a phenomenological approach will be used.

#### Population and sample

The sample will be purposive [61, 64, 77], favouring maximum variability in PCHP characteristics and those who place value on displays of gratitude. It is forecast that participants will be mainly doctors and nurses as these are the HP predominantly found in the teams and to whom the patients specifically direct their gratitude.

Approaches will be made to possible participants through the contacts facilitated on finalising the questionnaire, with a view to them continuing to take part in the study (first phase) and through the contacts, which the research team has of HP interested in gratitude displays. It is quite normal that in mixed method design, some of the participants in the quantitative phase also take part in the qualitative phase. When selecting possible interview participants that come from the quantitative part, it will be reviewed their answer to open questions (ie: describe a gratitude situation meaningful to you and describe why it was so special for you) and socio-demographic characteristics to enhance identifying rich informants with different characteristics and perspectives.

Potential participants will be contacted to invite them to participate in the interview, telling them of the implications of their taking part, confirming confidentiality as regards their details and requesting their informed consent. Saturation point wants to be achieved [78] so it is planned that in case no sufficient rich interviewees are obtained through the questionnaire and research team contacts (as mentioned above), further possible participants will be identified using snow-ball technique [64] with interview participants to identify further interviewees.

In qualitative research is difficult to specify the sample size required to achieve the objective. The phenomenological studies reviewed have used between 6 and 36 participants [79–83]. We estimate that 20 participants sample may be enough for saturation point; which is the point where no matter how much lived experiences continue collecting it will not be reached a greater or better understanding of the phenomenon studying [78].

#### Data collection

Individual conversational interviews will be carried out with PCHP. They will be asked about their individual experience and the significance of receiving displays of gratitude from P/R.

#### Guideline development and interview process

The interview guide will be designed taking the survey results and literature review into account. It is foreseen that HP will be encouraged to tell of their specific experiences of displays of gratitude from P/R they have cared for and to talk of what these displays actually meant to them, the significance for them and use they have made of the same.

The interviews will be conducted face-to-face or by video link, after earlier agreeing on the day and time with the interviewee. The interview will begin with a general question as an introduction and depending on the participants’ narrative, there will be a combination of scripted open questions, together with other dialogue questions, with the aim of widening the scope of the answers offered by the participants, allowing them to clarify their statements or to request specific examples of situations they have lived.

If needed for deepening or clarification, a further interview might be carried out with the HP to go into greater depth and to understand their experience [84].

Interviews will be audio recorded and then transcribed for analysis.

#### Pilot interviews

The lead researcher will conduct all the interviews (MAPA). Since personal interview skill development is essential, a number of pilot interviews will be conducted, for which feedback from an expert in interviews (MARANTZ) will be provided on how to encourage respondents to talk and to elicit experiential narrative material, stories or anecdotes, that may serve as a resource to develop a richer and deeper understanding of the gratitude phenomenon and how to use different probing strategies –if needed [85]. The purpose of the pilot is mainly to improve interviewer’s skills, who has already previous experience on conducting qualitative research so it is estimated that 2–3 interviews may be enough to adjust the interviewing skills to a phenomenological approach. The data of the pilot interviews will not be included in the data analysis.

#### Data analysis

Transcriptions will be literal, word for word, in order to maintain the richness of the data and encourage rigour. They will undergo phenomenological centred qualitative analysis [85], since this is suitable for describing factors common to experiences in HP, given that this method studies how people construct meaning from lived experiences [86]. To do this, specific themes from transcriptions will be taken separately and reflected upon on two levels: macro-thematically and micro-thematically to deep on the essence of the lived experience of receiving gratitude displays from P/R. Using macro-thematic reflection, after reading the text, one or more judgmental statements will be made regarding the fundamental meaning of the text (from the interview) or the importance of the same overall. Using this approach, we try to answer the questions, “What does this text say about the meaning of the phenomenon which we are studying?” “Which judgmental statement or statements might embody the fundamental meaning or the main idea behind the text overall?” [85, 87–89].

Then a micro-thematic reflection will be undertaken, on two levels [88]. To begin with, using the selective approach, the text will be read a number of times, considering which statement or statements seem to be especially important or revealing, on the phenomenon or experience being described. Then, those which seem to reveal a thematic aspect of the phenomenon will be highlighted. Secondly, using a detailed, or line-by-line approach, each statement or group of statements will be reviewed individually, whilst reflecting on what that statement or group of statements reveal about the experience of demonstrations of gratitude.

As the selective approach progresses, so will the process of beginning to edit those fragments which reveal any thematic aspect on the experience lived from gratitude, transcribing this content in literal fragments which may be used as examples of lived anecdotes.

The analytical process will be undertaken by one of the researchers (MAPA) under the constant supervision of an expert in qualitative research (MARANTZ). Throughout the analysis process, meetings will be held to reflect on the interpretative process and the results emerging with another PC expert (CCC).

### Ethical aspects

The Research Ethics Committee of the Universidad de Navarra approved the study with the number 2016.071.

In the study, an information sheet will inform all participants about the different phases of the project in which they are participating, explaining the context, aims, implications of taking part and the ethical aspects.

In both the quantitative and the qualitative study, participants will receive information on the implications of their taking part and the possibility of dropping out whenever they wish.

In the case of the survey it is assumed that by accepting to complete and send it, participants are giving their consent to take part of it. As for the interviews, an additional signed consent form will be required.

Norms regarding confidentiality and rigour in scientific research will be observed in handling personal details. Data from the questionnaires will be anonymous and the details for HP who agree to be contacted for the interview phase will be kept on a separate database from that for the questionnaire and will be confidential.

Interviewees will be identified by a code and only the research team will have access to the same. In case results are published, only individual fragments will be cited, in such a way that identification of the participants will not be possible.

As well as ensuring that all the data is stored securely, the study will be carried out in compliance of current legislation on data protection (LO 15/99 on *Protección de Datos de Carácter Personal y su Reglamento de Desarrollo*).

## Discussion

This study will tackle the gap identified in the literature. The literature reviewed suggests that displays of gratitude received from P/R seem to have a positive effect on HP [4]. This literature consists of individual reflections of HP who tell their experience and mention that receiving gratitude from P/R leads to feelings such as well-being [22], recognition [25, 27], satisfaction [22–25], motivation [23] and even personal and professional repercussions [25, 27, 28, 90]. The characteristics and impact of receiving gratitude have not been fully examined and this has never been done systematically in PC. HP in this area are especially exposed to situations of suffering and this makes research into the potentially beneficial effects of gratitude received from P/R especially interesting to offer a resource that can help us to carry on in our daily duty; even if we do not do our work expecting to receive gratitude.

The objective of this study is to understand the experience of gratitude received when caring and its significance for PCHP, by means of a mixed method research.

The use of a mixed method design provides a broad approach to explore the topic, which is adequate to explore a complex little known phenomenon. The first part of the study will allow the systematic study of a large sample of HP working on PC services and their experiences of gratitude. As any self-reporting study it has limitations regarding recall bias. Considering the literature about the possible effect of gratitude displays and the value that HP give to them (ie: they keep letters and gifts), this may be minor to other situations. The questionnaire includes open questions on which they describe experiences obtaining details that otherwise may be overlooked. It has been carefully planned to reach all the PC services, containing all the possible types of PC resources, as mentioned earlier, promoting representativeness. The member of the team that publicly appears like the service contact is the one that receives the questionnaire. This person is usually the head of the service so it is assumed that will have an overall view of the phenomenon. This view may differ from other members but allows to provide a general national view of the gratitude displays that PCHP receive as all the PC services are included. To our knowledge, this would be the first study to this regard with such a large coverage.

A careful plan has been developed to improve the response rate but in spite of this there will be non-respondents. It is not planned to gather information from these because it has been prioritized the objective of deepening on the gratitude experiences, developing the qualitative part of the study. In it, the results obtained in the survey will be taken into account but prioritizing in deepening in the lived experiences of the HP. It is planned to reduce the limitations of having only participants of the survey for the interviews, considering the inclusion of other participants interested in the subject. In studies with a phenomenological approach, the key to the quality of the study is not based as much on the number of participants as on the richness and depth of the data obtained. For this reason, a pilot phase has been designed to refine the skills of the interviewer and carry out a quality study advised by an expert. Besides, different measures have been planned to assure enough interviewees to reach saturation.

The quantitative part will provide an initial examination of the phenomenon, by means of a nationwide survey, to obtain general information on the subject and to evaluate the true situation of displays of gratitude in PC and their self-reported repercussion. The qualitative part will add deeper insight on the significance and role that displays of gratitude received from P/R have for PCHP.

This study has the strength to demonstrate with a national coverage, the wider view about the characteristics of the gratitude displays received in PC services from P/R, and the meaning, impact and significance that this gratitude has for the PCHP.

This study contribution could be important within the context of the growing preoccupation on how to care for HP, how to ensure retention and keep them on as part of the teams at institutions, how to prevent burnout, fatigue issues and loss of motivation, which might give rise not only to impersonal care but also to increased absenteeism at work. If the results confirm the positive effects mentioned anecdotally in literature, this will give rise to an interesting field of study on expressions of gratitude as a source of resources to tackle burnout or to enhance resilience.

## Additional file

Additional file 1: Questionnaire: phase one of the study. (DOCX 92 kb)

## Abbreviations

CCC: Carlos Centeno Cortés
HP: Healthcare professional
MAPA: Maria Aparicio
MARANTZ: María Arantzamendi
P/R: patient / relatives
PC: Palliative care
PCHP: Palliative care health professional

## References

[1] McCullough ME, Kilpatrick SD, Emmons RA, Larson DB (2001). Is gratitude a moral affect?. Psychol Bull.

[2] Simão C, Seibt B. Gratitude depends on the relational model of communal sharing. PLoS One. 2014;9(1). 10.1371/journal.pone.0086158 PMC3899114 24465933

[3] Schwarz B (2004). Del Agradecimiento.

[4] M. Aparicio, C. Centeno, C. Robinson, and M. Arantzamendi, Gratitude between patients and their families and health professionals : A scoping review. J Nurs Manag, no. April, pp. 1–15, 2018. 10.1111/jonm.12670 30084234

[5] R. A. Emmons, Gratitude, subjective well-being, and the brain in *The science of subjective well-being.*, M. Eid and R. J. Larsen, Eds. NY: Guilford Press, 2008, p. 546.

[6] Emmons RA, McCullough ME (2003). Counting blessings versus burdens: an experimental investigation of gratitude and subjective well-being in daily life. J Pers Soc Psychol.

[7] Wood AM, Froh JJ, Geraghty AWA (2010). Gratitude and well-being: a review and theoretical integration. Clin Psychol Rev.

[8] R. Roberts, The Blessings of Gratitude: a conceptual Analysis in *The phycology of the Gratitude*, Oxford., R. A. & Emmons and M. E. McCullough, Eds. Oxford University Press, 2004, p. 368.

[9] Elosúa MR (2015). The influence of gratitude in physical, psychological, and spiritual well-being. J Spiritual Ment Heal.

[10] Lai ST. The efficacy of gratitude practice on well-being: a randomized controlled trial (master thesis): University of Stirling; 2014.

[11] Sansone RA, Sansone LA (2010). Gratitude and well being: the benefits of appreciation. Psychiatry.

[12] Watkins PC, Woodward K, Stone T, Kolts RL (2003). Gratitude and happiness: development of a measure of gratitude, and relationships with subjective well-being. Soc Behav Personal Int J.

[13] Krause N (2006). Gratitude toward god, stress, and health in late life. Res Aging.

[14] K. Tsui Pui, Gratitude and stress of health care professionals in Hong Kong,” City University of Hong Kong, 2009.

[15] Wood AM, Joseph S, Maltby J (2008). Gratitude uniquely predicts satisfaction with life: incremental validity above the domains and facets of the five factor model. Pers Individ Dif.

[16] Lau RWL, Cheng ST (2011). Gratitude lessens death anxiety. Eur J Ageing.

[17] Chan DW (2011). Burnout and life satisfaction: does gratitude intervention make a difference among Chinese school teachers in Hong Kong?. Educ Psychol.

[18] Lanham ME, Rye MS, Rimsky LS, Weill SR (2012). How gratitude relates to burnout and job satisfaction in mental health professionals. J Ment Heal Couns.

[19] Sansó N, Galiana L, Oliver A, Pascual A, Sinclair S, Benito E (2015). Palliative care professionals’ inner life: exploring the relationships among awareness, self-care, and compassion satisfaction and fatigue, burnout, and coping with death. J Pain Symptom Manag.

[20] Slocum-Gori S, Hemsworth D, Chan WW, Carson A, Kazanjian A (2011). Understanding compassion satisfaction, compassion fatigue and burnout: a survey of the hospice palliative care workforce. Palliat Med.

[21] B. Fredrickson, Gratitude and other Positive Emotions Broaden and Build. in *The Phycology of the gratitude*, R. A. Emmons and M. E. McCullough, Eds. 2004.

[22] Bollinger E (2001). Applied concepts of holistic nursing. J Holist Nurs.

[23] Evans H (2014). To Refuse Gifts from Patients would be Rude and Churlish. Nurs. Stand.

[24] Owen S (2002). A new year for Emily. Mich Nurse.

[25] Romanzini EM, Bock LF (2010). Conceptos y sentimientos de enfermeros que actúan en la atención pre-hospitalaria sobre la práctica y la información profesional. Rev Lat Am Enferm.

[26] J. Zabresky, “Hey Nurse...,” *Nursing (Lond).*, vol. August, p. 49, 1993. 10.1097/00152193-199308000-00023 8361698

[27] Raingruber B, Wolf T (2015). Nurse perspectives regarding the meaningfulness of oncology nursing practice. Clin J Oncol Nurs.

[28] D. Converso, B. Loera, S. Viotti, and M. Martini, Do positive relations with patients play a protective role for healthcare employees? Effects of patients’ gratitude and support on nurses’ burnout. Front. Psychol*.* vol. 6, no. April, pp. 1–11, 2015. 10.3389/fpsyg.2015.00470 PMC4404725 25954227

[29] Lissandre S, Abbey-Huguenin H, Bonnin-Scaon S, Arsene O, Colombat P. Facteurs associés au burnout chez les soignants en oncohématologie. Oncologie. 2008.

[30] Martins Pereira S, Fonseca AM, Sofia Carvalho A (2011). Burnout in palliative care: a systematic review. Nurs Ethics.

[31] Kamal AH, Bull JH, Wolf SP, Swetz KM, Shanafelt TD, Ast K, Kavalieratos D, Sinclair CT, Abernethy AP. Prevalence and predictors of burnout among hospice and palliative care clinicians in the U.S. J. Pain Symptom Manage. 2016. 10.1016/j.jpainsymman.2015.10.020 PMC4846384 26620234

[32] Martínez García M, Centeno Cortés C, Sanz Rubiales A, Del Valle ML (2009). Estudio sobre el Síndrome de Burnout en Profesionales de Enfermería de Cuidados Paliativos del País Vasco. Rev Med.

[33] Peters L, Cant R, Sellick K, O’Connor M, Lee S, Burney S (2012). Is work stress in palliative care nurses a cause for concern? A literature review. Int J Palliat Nurs.

[34] Joinson C (1992). Coping with compassion Fatique. Nursing (Lond).

[35] R. Campos Méndez, “Estudio sobre la prevalencia de la fatiga de la compasión y su relación con el síndrome de ‘burnout’ en profesionales de Centros de mayores en Extremadura,” p. 418, 2015.

[36] M. del C. Hernández García, Fatiga por compasión entre profesionales sanitarios de oncología y cuidados paliativos. Psicooncologia, vol. 14, no. 1, pp. 53–70, 2017.

[37] Sinclair S, Raffin-Bouchal S, Venturato L, Mijovic-Kondejewski J, Smith-MacDonald L (2017). Compassion fatigue: a meta-narrative review of the healthcare literature. Int J Nurs Stud.

[38] Lefton C (2012). Strengthening the workforce through meaning recognition. Nurs Econ.

[39] A. American Association of Critical-Care Nurses, “AACN Standars for Stablishing and Sustaining Healthywork Environments: a Journey to Excellence,” vol. 14, no. 3, pp. 187–197, 2005. 15840893

[40] Moran D, Elliott R, McKinley S (2005). The Royal North Shore Hospital ICU nurse initiated telephone follow up service. Intensive Crit. Care Nurs..

[41] McLaughlin D, Sullivan K, Hasson F (2007). Hospice at home service: the carer’s perspective. Support Care Cancer.

[42] Meert KL, Eggly S, Pollack M, Anand KJS, Zimmerman J, Carcillo J, Newth CJL, Dean JM, Willson DF, Nicholson C (2007). Parents’ perspectives regarding a physician-parent conference after their Child’s death in the pediatric intensive care unit. J Pediatr.

[43] Engström Å, Andersson S, Söderberg S (2008). Re-visiting the ICU. Experiences of follow-up visits to an ICU after discharge: a qualitative study. Intensive Crit Care Nurs.

[44] Barry CD, Lange B, King B (2011). Women alive: gathering underserved women upstream for a comprehensive breast health program. South Online J Nurs Res.

[45] Ekblad S, Linander A, Asplund M (2012). An exploration of the connection between two meaning perspectives: an evidence-based approach to health information delivery to vulnerable groups of Arabic- and Somali-speaking asylum seekers in a Swedish context. Glob Health Promot.

[46] Tranter S, Anastasiou A, Bazzi H, Burgess H, Josland E (2013). The renal memorial service: an important component of renal supportive care. Ren Soc Australas J.

[47] Rahmani Z, Brekke M (2013). Antenatal and obstetric care in Afghanistan - a qualitative study among health care receivers and health care providers. BMC Health Serv Res.

[48] Cacciatore J, Erlandsson K, Rådestad I (2013). Fatherhood and suffering: a qualitative exploration of Swedish men’s experiences of care after the death of a baby. Int J Nurs Stud.

[49] M. Arantzamendi and C. Centeno, Intangible values of palliative care. Eur. J. Palliat. Care, vol. (24), no. 2, pp. 72–74, 2017.

[50] Aparicio M. A Satisfação dos Familiares de Doentes em Cuidados Paliativos (Master thesis): Faculdade de Medicina de Lisboa; 2008.

[51] Begg TA (1994). Give and You Shall Receive. Am. J. Nursisng.

[52] Hernandez-Marrero P, Pereira SM, Carvalho AS (2016). Ethical decisions in palliative care: Interprofessional relations as a burnout protective factor? Results from a mixed-methods multicenter study in Portugal. Am J Hosp Palliat Med.

[53] Creswell J, Klassen AC, Plano V, Smith KC (2011). Best practices for mixed methods research in the health sciences. Methods.

[54] Polit DF, Beck CT (2014). Essentials of nursing research: appraising evidence for nursing practice.

[55] Creswell JW (2003). Research design: qualitative quantitative and mixed methods approaches.

[56] Creswell JW (2014). Research Desing. Qualitative, Quantitative and Mixed Methods Approaches.

[57] Tashakkori A, Teddlie C. Mixed methodology: combining qualitative and quantitative approaches. London; 1998.

[58] Moran-Ellis J. Triangulation and integration: processes, claims and implications: Qual. Res; 2006.

[59] Oriol I (2014). Informe De La Situación Actual En Cuidados Paliativos.

[60] Doblado R, Herrera E, Librada S, Lucas MÁ, Muñoz I, Rodríguez Z. SECPAL-Directorio de Recursos de Cuidados Paliativos en España: SECPAL; 2015.

[61] Fortin M-F (2009). Fundamentos e etapas do processo de investigação.

[62] McColl E, Jacoby A, Thomas L, Soutter J, Bamford C, Steen N (2001). Design and use of questionnaires:a review of best practice applicable to surveys of health service staff and patients. Health Technol Assess (Rockv).

[63] A. Fink, “How to sample in surveys,” SAGE publications, Ed. California, 2003.

[64] Kelley K, Clark B, Brown V, Sitzia J (2003). Good practice in the conduct and reporting of survey research. Int J Qual Heal Care.

[65] Vidal Díaz de Rada, “Ventajas e inconvenientes de la de la encuesta por Internet,” *Papers*, vol. 97/1, pp. 193–223, 2012.

[66] B. Poole and D. Loomis, “A comparative analysis of mail and internet surveys,” Proc. 2009 Northeast. Recreat. Res. Symp*.*, pp. 231–234, 2009.

[67] Cook C, Heath F, Thompson R (2000). A meta-analysis of response rates in web-or internet-based surveys. Educ Psychol Meas.

[68] Sánchez-Fernández J, Muñoz-Leiva F, Montoro-Ríos FJ (2009). Cómo Mejorar La Tasa De Respuesta En Encuesta on Line?. Rev Estud Empres.

[69] P. M. Boynton, Administering, analysing, and reporting your questionnaire. BMJ Br. Med. J*.*, vol. 328, no. June, pp. 1372–1375, 2004. 10.1136/bmj.328.7452.1372 PMC420299 15178620

[70] Sitzia J, Wood N (1998). Response rates in patient satisfaction research: an analysis of 210 published studies. Int J Qual Heal Care.

[71] K. I. M. B. Sheehan, J. August, J. Of, and R. Reserrcil, Response Variation in E-iVlaii Surveys : An Expioration, J. Advert. Res*.* vol. July-Augus, 1999.

[72] Cobanoglu C, Warde B, Moreo PJ (2001). A comparison of mail, fax and web-based survey methods. Int. J. Mark.

[73] S. N. Hoddinott and M. J. Bass, The Diliman Total Design Survey Method : A Sure-Fire way to Get High Survey Return Rates. Can. Fam. Physicians, vol. 32, no. November, pp. 2366–2368, 1986. PMC2328022 21267217

[74] Edwards P, Roberts I, Clarke M, DiGuiseppi C, Pratap S, Wentz R, Kwan I (2002). Increasing response rates to postal questionnaires: systematic review. BMJ.

[75] Dilman DA. Introduction to Tailored Design. 2nd ed. New York; 2007.

[76] S. A. Gordon, Susan E: Stokes, Improving Response Rates to Mailed Questionnaires. Nurs. Res., vol. 38, no. n^o^6, pp. 375–376, 1989. 2587295

[77] Hicks CM (2006). Métodos de Investigação para Terapeutas Clinicos, 3^a^.

[78] S. M. Laverty, Hermeneutic Phenomenology and Phenomenology: A Comparison of Historical and Methodological Considerations. Int. J. Qual. Methods, vol. 2, no. September, pp. 1–29, 2003.

[79] C. C. Chao and C. C. Miaofen, “The Essence of Spir i tu al ity of Ter mi nally I ll Patients,” vol. 10, no. 4, pp. 237–245, 2002.

[80] McGrath P, Moore A, McNaught M, Palmer G, Greene A, Atkinson D (2005). Another form to fill in! Clients’ reflections on the hospice use of questionnaires. Support Care Cancer.

[81] Pusa S, Hägglund K, Nilsson M, Sundin K (2015). District nurses’ lived experiences of meeting significant others in advanced home care. Scand J Caring Sci.

[82] Marrero CM, García AM (2017). La vivencia del paso de estudiante a profesional en enfermeras de tenerife (España). Un estudio fenomenológico. Rev ENE Enfermería.

[83] Gálvez González M, Ríos Gallego F, Fernández Vargas L, del Águila Hidalgo B, Muñumel Alameda G, Fernández Luque C (2011). El final de la vida en la Unidad de Cuidados Intensivos desde la perspectiva enfermera: Un estudio fenomenológico. Enferm Intensiva.

[84] Streubert HJ, Rinaldi Carpenter D (2013). Investigação qualitativa em enfermagem.

[85] Van Manen M. Phenomenology of practice : meaning-giving methods in phenomenological research and writing, vol. 2014. Walnut Creek, California, Left Coast Press, cop; 2014.

[86] Starks H, Brown Trinidad S (2007). Choose your method: a comparison of phenomenology, discourse analysis, and grounded theory. Qual Health Res.

[87] Van Manen M (2003). Investigación educativa y experiencia vivida : ciencia humana para una pedagogía de la acción y la sensibilidad.

[88] Errasti Ibarrondo MB. La Relación Interpersonal Entre La Enfermera Y La Persona Cuidada: Una Aproximación Desde La Experiencia Vivida De La Persona Con Cáncer En Fase Avanzada Y Terminal: University of Navarra; 2015.

[89] M. van Manen, “Phenomenology Online » Macro-thematic Reflection,” 2017. [Online]. Available: http://www.phenomenologyonline.com/inquiry/methods-procedures/reflective-methods/thematic-reflection/macro-thematic-reflection/. [Accessed: 12-Jun-2017].

[90] A. L. Jones, The Rewards of Rehabilitation Nursing- A Story of Gratitude. Rehabil. Nurs. Off. J. Assoc. Rehabil. Nurses, vol. 30, no. 2, p. 39, 2004. 10.1002/j.2048-7940.2005.tb00356.x 15789693

