# Supplementary material for: The significance of gratitude for palliative care professionals: a mixed method protocol
Source: BMC Palliat Care. 2019 Mar 21;18:28. doi: 10.1186/s12904-019-0412-y (PMC6427884; doi:10.1186/s12904-019-0412-y)
Supplement: Supplementary file 1 — Questionnaire: phase one of the study. (DOCX 92 kb) [file 12904_2019_412_MOESM1_ESM.docx]

**THE EXPERIENCE OF GRATITUDE WHEN CARING AND ITS MEANING TO PALLIATIVE CARE PROFESSIONALS**

**PART ONE**

**We would like to explore some aspects related to the experience of receiving gratitude in your palliative care service.**

1. How **often** do patients or their relatives show their gratitude in your workplace?

Please choose one option:


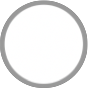
Almost never


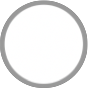
Sometimes


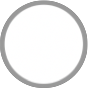
Regularly


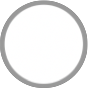
Very often


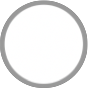
Nearly always

1. Which **displays of gratitude** from patients or relatives are received most frequently in your workplace?

Please choose one answer for each of the options:

|  | Almost never | Sometimes | Regularly | Very often | Nearly always |
| --- | --- | --- | --- | --- | --- |
| Words of thanks |  |  |  |  |  |
| Letters of thanks |  |  |  |  |  |
| Cards or post cards |  |  |  |  |  |
| Greetings cards on special occasions (Christmas, Easter, Anniversaries, etc.) |  |  |  |  |  |
| Plaques with messages of gratitude |  |  |  |  |  |
| Flowers |  |  |  |  |  |
| Gifts of foods |  |  |  |  |  |
| Personal gifts |  |  |  |  |  |
| Donations |  |  |  |  |  |

Others (specify)

1. **Which people** show their gratitude most often in your workplace?

Please choose one answer for each of the options:

|  | Almost never | Sometimes | Regularly | Very often | Nearly always |
| --- | --- | --- | --- | --- | --- |
| The patient |  |  |  |  |  |
| The patient's family |  |  |  |  |  |
| Friends of the patient |  |  |  |  |  |

Others (specify)

1. In which **moments** are displays of gratitude received most frequently in your workplace?

Please choose one answer for each of the options:

|  | Almost never | Sometimes | Regularly | Very often | Nearly always |
| --- | --- | --- | --- | --- | --- |
| On first meeting them |  |  |  |  |  |
| Throughout the care process |  |  |  |  |  |
| In the patient's final moments |  |  |  |  |  |
| Immediately after the patient's death |  |  |  |  |  |
| During bereavement |  |  |  |  |  |
| A long time after the care relationship has finished |  |  |  |  |  |

Others (specify)

1. When documents expressing gratitude are received (letters, cards or postcards) from patients and their relatives in your workplace, **What use** is most often made of them?

Please choose one answer for each of the options:

|  | Almost never | Sometimes | Regularly | Very often | Nearly always |
| --- | --- | --- | --- | --- | --- |
| Some members of the team read them |  |  |  |  |  |
| They are on display for all members of the team |  |  |  |  |  |
| They are kept for some time |  |  |  |  |  |
| Some team members keep them among their personal effects |  |  |  |  |  |
| They are filed away and are accessible to all team members |  |  |  |  |  |
| They are filed away but are not accessible |  |  |  |  |  |
| They are sent to management |  |  |  |  |  |
| They are sent to other team members who have taken part in the care process |  |  |  |  |  |

Other (specify)

1. **What feelings** arise when displays of gratitude by patients or relatives are received in your service?

Please choose the 5 most frequent answers:


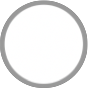
Motivation


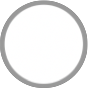
Satisfaction


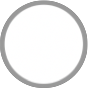
Personal fulfilment


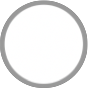
Sadness


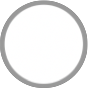
Pride in my work


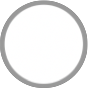
Emotional exhaustion


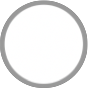
Well-being


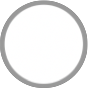
Gratitude


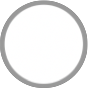
Joy


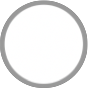
Malaise


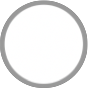
Others (specify)

**PART TWO**

**Below you will find questions related to your personal experience, so we would appreciate your responses from a personal perspective.**

**Think of a special situation in which you received thanks from a patient or a relative and which was meaningful to you.**

1. Could you give a brief **description** of this situation?

(What type of display, who was it from, why did it happen...?) Maximum 200 words.

8. Concerning the situation you describe. **Why** was it so **special or meaningful**? (What meaning did it hold for you...?) Maximum 200 words.

1. As a palliative care professional, **what role does gratitude** from patients or relatives play in your professional practice?

Please choose the degree to which you agree with the following statements, choosing one option for each:

|  | Strongly disagree |  |  |  | Strongly Agree | |  |
| --- | --- | --- | --- | --- | --- | --- | --- |
|  | 1 | 2 | 3 | 4 | | 5 | |
| Receiving gratitude increases satisfaction at work |  |  |  |  | |  | |
| Receiving gratitude increases professional commitment |  |  |  |  | |  | |
| Receiving gratitude makes one feel more fulfilled professionally |  |  |  |  | |  | |
| Receiving gratitude makes one feel more fulfilled personally |  |  |  |  | |  | |
| Receiving gratitude improves one's professional state of mind |  |  |  |  | |  | |
| Receiving gratitude is a source of support in professionally difficult moments |  |  |  |  | |  | |
| Receiving gratitude helps reflect on care given |  |  |  |  | |  | |
| Receiving gratitude encourages one to go on in palliative care |  |  |  |  | |  | |
| Receiving gratitude makes up for the efforts given |  |  |  |  | |  | |
| Receiving gratitude helps find meaning to work done |  |  |  |  | |  | |
| Receiving gratitude stimulates more compassive care |  |  |  |  | |  | |
| Receiving gratitude reduces burnout* |  |  |  |  | |  | |
| Receiving gratitude protects against compassion fatigue** |  |  |  |  | |  | |

*Burnout is defined as a syndrome, characterised by the existence of emotional weariness, depersonalization and low personal fulfilment, which may be evident in professionals who carry out any kind of work involving attention to other people (Maslach and Jackson, 1981).

**Compassion Fatigue is defined as stress resulting from helping or wanting to help a person who is traumatised or who is suffering (Figley, 1982).

**PART THREE**

**To finish, we would like to ask you some questions related to your sociodemographic characteristics.**

- 10. **Sex**. Please choose one option:
-
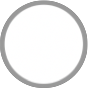
Male


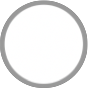
Female

- 11. **Age**. Please show your age in years:
- 12. **Marital status**. Please choose one option:
-
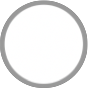
Married
-
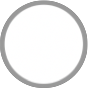
Civil union
-
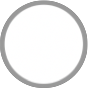
Single
-
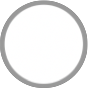
Separated


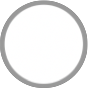
Divorced

-
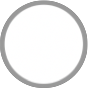
Widowed
-
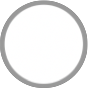
Other (specify):

13. Have you **lost a close family member** in the last 12 months? Please choose one option:


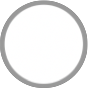
Yes


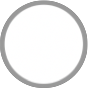
No

14. **Profession**. Please choose one option:


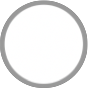
Doctor


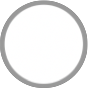
Nurse


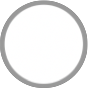
Psychologist


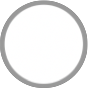
Social worker


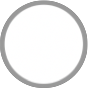
Physiotherapist


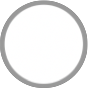
Occupational therapist


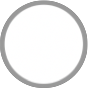
Health Care Assistant


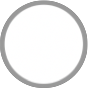
Chaplain or faith worker


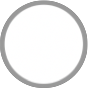
Other (specify):

15. Years of experience **dedicated full time** to palliative care

Please indicate your experience, in years:

**16.** Indicate the **type of palliative care service** where you work

Please choose one option:


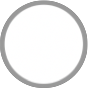
Hospital Palliative Care support team


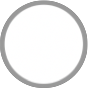
Home based Palliative Care support team


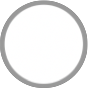
Mixed (home based and hospital) Palliative Care support team


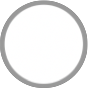
Palliative Care Unit


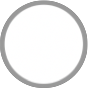
Hospice


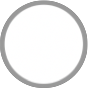
Hospital Paediatric Palliative Care team


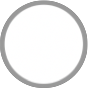
Home based Paediatric Palliative Care team


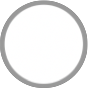
Paediatric Palliative Care unit


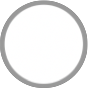
Other (specify):

17. **Number of professionals** who make up the palliative care service where you work.

Please choose one option:


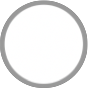
Less than 6


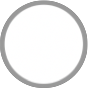
Between 6 and 12


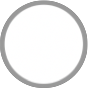
More than 12

18. **Region** where you work

Please press and choose one option:


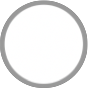
Andalusia


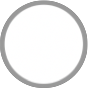
Aragón


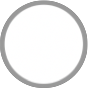
Canary Islands


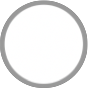
Cantabria


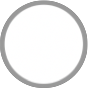
La Mancha


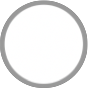
Castile and Leon


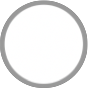
Catalonia


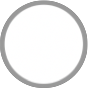
Navarre


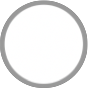
Madrid


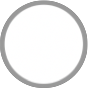
Valencia


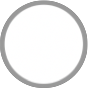
Extremadura


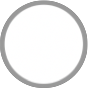
Galicia


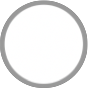
Balearic Islands


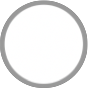
Rioja


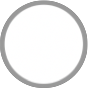
Basque Country


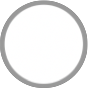
Asturias


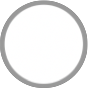
Murcia


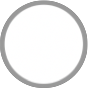
Ceuta


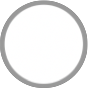
Melilla

19. **Name of the Palliative Care service** where you work:

**In order to obtain more details on displays of gratitude by patients or relatives, we would like to be able to contact more palliative care professionals from other services.**

20. Would you be able to suggest the **name and e-mail contact** of 2 people from different palliative care service who might be interested in contributing to this study (Optional response)

**Name**

**Email**

**Name**

**Email**

**In the second part of our study, we shall carry out interviews with palliative care professionals. We are looking for professionals who appreciate or realise the importance of receiving displays of gratitude.**

21. If you would like to take **part in one of these interviews**, please provide your details (Optional response):

**Name Email**

**Telephone**

**Best day of the week for contact**

**Best time of day for contact**
